# Electroencephalographic Aspects in a Sheep with Coenurosis

**DOI:** 10.3390/life16091504

**Published:** 2026-09-09

**Authors:** Paula Maria Pașca, Gheorghe Solcan, Raluca Adriana Ștefănescu, Iordana Stoica, Sorin Aurelian Pașca, Loredana-Elena Olar, Vasile Daniel Tomoiaga, Caroline-Maria Lacatus, Robert Cristian Purdoiu, Radu Lăcătuș, Mihai Musteata

**Affiliations:** 1 Clinics Department, Faculty of Veterinary Medicine, Ion Ionescu de la Brad Iași University of Life Sciences, 700489 Iași, Romania; paula.pasca@iuls.ro (P.M.P.); raluca.stef@yahoo.ro (R.A.Ș.); iordana_stoica@yahoo.com (I.S.); mihai.musteata@iuls.ro (M.M.); 2 Department of Pathological Anatomy, Faculty of Veterinary Medicine, Ion Ionescu de la Brad Iași University of Life Sciences, Mihail Sadoveanu Alley, 700489 Iași, Romania; sorin.pasca@iuls.ro; 3 Faculty of Veterinary Medicine, University of Agricultural Sciences and Veterinary Medicine, 400372 Cluj-Napoca, Romania; loredana.olar@usamvcluj.ro (L.-E.O.); vasile.tomoiaga@usamvcluj.ro (V.D.T.); carolinelacatus@yahoo.com (C.-M.L.); robert.purdoiu@usamvcluj.ro (R.C.P.); radu.lacatus@usamvcluj.ro (R.L.); 4 Life Sciences Institute, 3-5 Manastur Avenue, 400372 Cluj-Napoca, Romania

**Keywords:** ovine coenurosis, electroencephalography, brain cyst, coenurosis diagnostic

## Abstract

Ovine coenurosis, caused by the larval stage of *Taenia multiceps* (*Coenurus cerebralis*), is a parasitic disease that affects the central nervous system of livestock. While diagnosis traditionally relies on clinical signs, imaging, and postmortem examination, electroencephalography (EEG) remains underutilized despite its potential to detect brain abnormalities. This case report describes EEG and quantitative EEG (qEEG) findings in a 2-year-old sheep presenting with clinical signs suggestive of a chronic focal encephalopathy (ataxia, head tilt and circling) associated with lethargy and progressive weight loss. Computed tomography (CT) confirmed a single, discretely bilobate intra-axial cystic lesion within the left cerebral hemisphere, with a severe mass effect, marked deformation and displacement of the ventricular system, compression of the thalamic structures, and contralateral deviation of the median line. A solitary parasitic cyst with no lesion in the right hemisphere was subsequently confirmed at necropsy and on histopathology. Under ketamine–diazepam sedation, both EEG recordings showed a continuous, delta-dominant background consistent with the anesthetic protocol. Epoch-wise asymmetry indices with bootstrap confidence intervals distinguished an unstable component (which reversed in hemispheric sign between recordings) from a reproducible one confined to the occipital derivations, where the asymmetry lay over the right hemisphere in both recordings in every band except delta. This reproducible preponderance was contralateral to the cyst, over a hemisphere in which no structural lesion was demonstrable either on imaging or at necropsy, and absolute powers indicated that it arose principally from attenuation of the signal recorded over the diseased hemisphere. These findings provide the first electrophysiological characterization of ovine coenurosis and show that in the presence of a severe mass effect, the side of the surface qEEG abnormality may not indicate the side of the lesion (an electrophysiological false localizing sign described in human patients but not previously in a ruminant). EEG therefore reflects the functional brain disturbance caused by *Coenurus cerebralis* but should be regarded as a complement to, rather than a substitute for, cross-sectional imaging in neurolocalization.

## 1. Introduction

Ovine coenurosis is a parasitic disease caused by the *Taenia multiceps* tapeworm (larval stage *Coenurus cerebralis*), a cestode which usually affects the central nervous system (CNS) of livestock [1,2]. Larval invasion of the central nervous system and clinical disease occur when pastures grazed by sheep are contaminated with dog feces that carry the parasite [2,3].

The disease may evolve in an acute or chronic presentation. The acute phase usually occurs in young animals and the clinical symptomatology (listlessness, pyrexia, head tilt or head pressing, convulsion and even death within 4 to 5 days) is the expression of the rapid and massive migration of the larval stage (oncospheres) of the tapeworm into the nervous tissue [3,4,5]. The chronic phase, more frequently observed, begins 4–6 months after the infestation and is a consequence of cyst development and subsequent increase in intracranial pressure [3,5]. Chronic coenurosis induces behavioral changes, postural deficits, circling, incoordination, and cranial nerve deficits [3,4].

The diagnosis of coenurosis in sheep traditionally relies on clinical signs, imaging techniques such as computed tomography (CT) or magnetic resonance imaging, and postmortem examination. In this way, the cyst’s dimension, localization, and impact on the cerebral parenchyma can be evaluated [3,5,6,7]. However, both advanced imaging and necropsic findings provide information only on lesion extension and are limited by their lack of information on the functional integrity of the cerebral parenchyma. By assessing the bioelectric activity using the EEG technique, subtle functional changes may be detected early.

In dogs, cystic lesions or pathologic enlargement of the ventricular system (such as hydrocephaly) may be investigated by the EEG technique; in this way, it is possible to identify subtle epileptic foci before the start of epileptic clinical activity. It is also possible to confirm neurolocalization and assess the evolution of the disease. For example, EEG failed to identify the location of cystic lesions but revealed the presence of epileptic discharges—such as spikes and spike–wave complexes—in an affected dog [8]. In hydrocephaly, EEG traces show the presence of diffuse high-amplitude low-frequency activity [9]. In sheep with coenurosis, brain electric activity was reported with scarcity. In another report [10], the presence of the coenurotic cyst in sheep was associated with altered electric brain activity. However, that report failed both in identifying the location of the cyst and in identifying a specific pattern when compared with other encephalopathies.

The aim of this study is to evaluate and describe the brain’s electric activity through EEG in a sheep with coenurosis, focusing on CT neurolocalization and histopathological features.

## 2. Case Description

A 2-year-old sheep was presented at the Veterinary Teaching Hospital of the Faculty of Veterinary Medicine in Iași, Romania, with lethargy, progressive weight loss, ataxia, head tilt, and loss of herd instinct. At admission, the physical examination revealed a body condition score of 2/5 and a normal respiratory frequency (19 resp./min) with a normal respiratory pattern. Upon auscultation, tachycardia (83 beats/min) was detected with a regular rhythm and no murmurs, and the results of lung auscultation were unremarkable. Mucous membranes were slightly pale, with capillary refill time < 2 s and rumen turnover 1/2 min. The subject’s rectal temperature was 38 °C, the perineal staining score was <2, and the mobility score was 1.

The patient was vaccinated against anthrax but had not received any antiparasitic treatment in the past year. No other previously known pathologies were reported, and no treatments or supplements were given prior to admission.

On neurological examination, the sheep showed an altered mental status, being disoriented and confused: it engaged in dromomania and was unable to overcome simple obstacles. For example, it failed to find its way through an open doorway and exhibited circling to the right and a right-sided head tilt. Cranial nerve assessment disclosed an absent menace response on the right and a diminished blink reflex on the right, elicited from both the medial and the lateral canthus and on stimulation of the facial skin and the pinna; the response to nasal (septal) stimulation was diminished on the right. Postural reactions were abnormal on the right side, with absent conscious proprioception in the right pelvic limb, reduced conscious proprioception in the right thoracic limb, and an altered hopping response in the right thoracic limb. The spinal reflexes were within normal limits, and the contralateral (left) cranial nerves and postural reactions were preserved.

A multifocal, left-lateralized forebrain and right brainstem neurolocalization was suspected, and an inflammatory infectious/parasitical etiology was privileged.

Blood samples were collected from the jugular vein to perform a complete blood count (CBC) and serum biochemistry. The CBC (Abaxis VetScan HM5-Zoetis Services, Parsippany, NJ, USA), showed mild normocytic anemia, with a red blood cell count of 8.47 × 10^12^/L [reference interval (RI) 8.5–9 × 10^12^/L], a hematocrit of 25.18% (RI 27–45%), and a hemoglobin level of 9.5 g/dL (RI 9–15 g/dL). The serum biochemistry (BA 200 analyzer—Biosystems, Barcelona, Spain) revealed low creatinine 0.66 mg/dL (RI 1.2–1.9 mg/dL), phosphate 4.01 mg/dL (RI 5–7.3 mg/dL), total proteins 53 g/L (RI 60–79 g/L), calcium 9.54 mg/dL (RI 11.5–12.8 mg/dL), and elevated alkaline phosphatase 548 U/L (RI 70–390 U/L), ALAT 43 U/L (RI 26–34 U/L), CK 712 U/L (RI 60–280 U/L) and glucose 91 mg/dL (RI 50–80 mg/dL).

**Figure 1:**
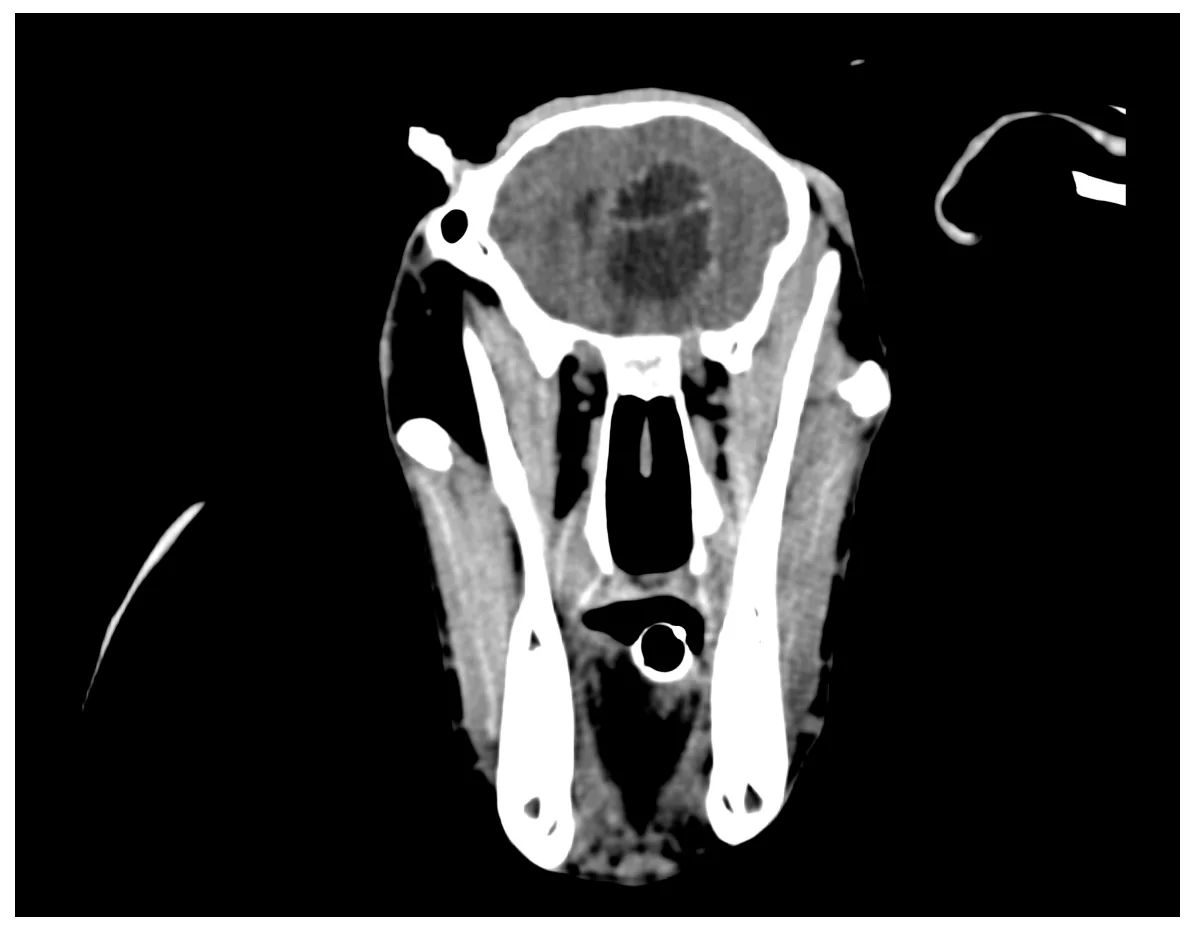
Helical CT examination; axial projection showing lightly bilobate intra-axial cystic/cavitary formation with mass effect on the adjacent structures.

**Figure 2:**
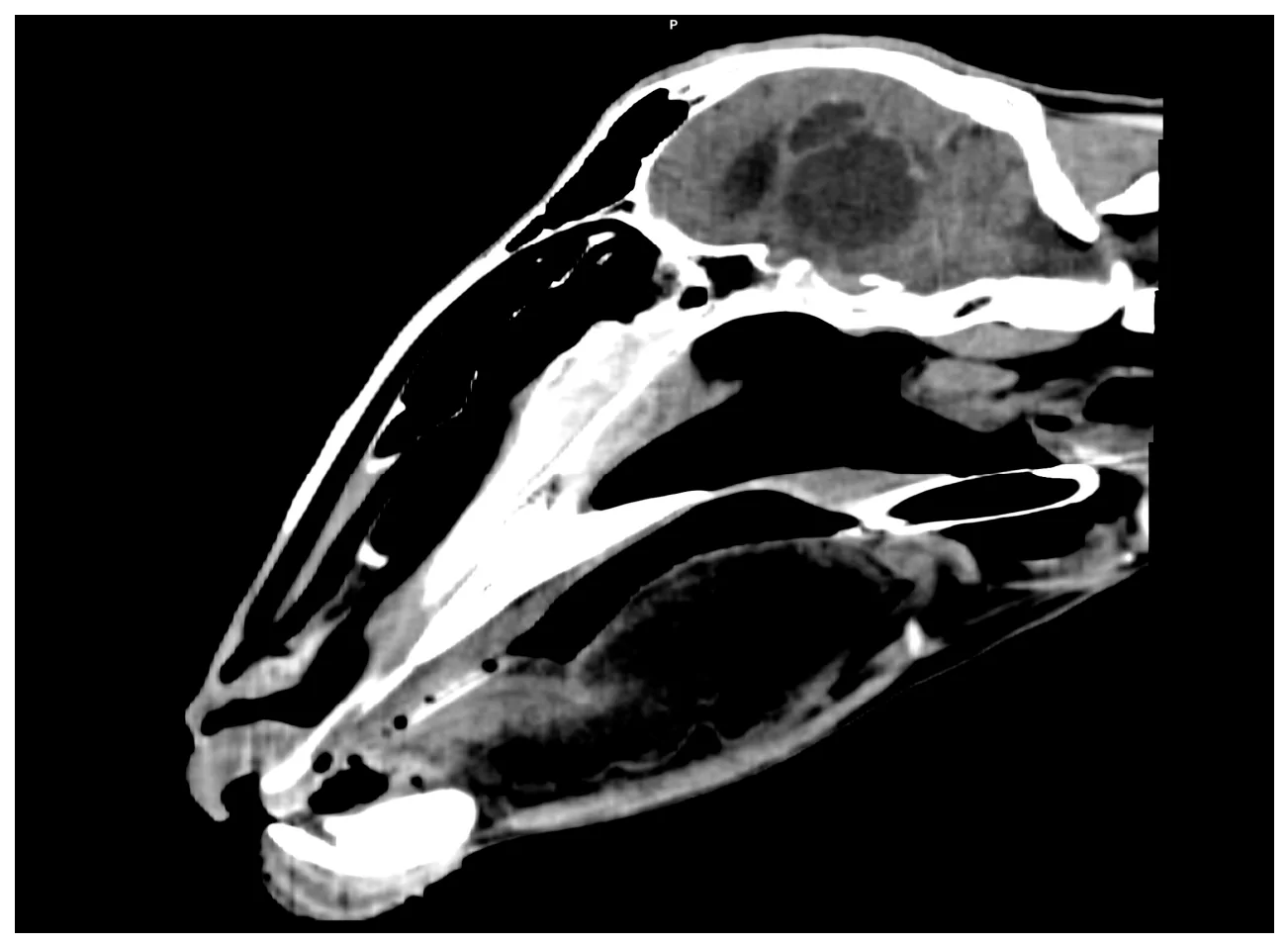
Helical CT examination; sagittal projection showing lightly bilobate intra-axial cystic/cavitary formation with mass effect on the adjacent structures.

CT examination of the head was performed in sternal recumbency, using a multidetector 16-slice CT scanner (Siemens Somatom Scope, Munich, Germany) in helical scan mode with the following parameters: 130 kVp, 200 mAs, and 1.25 slice thickness. Pre- and postcontrast delayed-phase examination was performed using a power injector (Mallinckrodt LF Dual Head CT Injector Optistat with OptiBolus, Dublin, Ireland) to infuse a bolus of 1.5 mL/kg of iodinated non-ionic contrast medium (Iohexol, Omnipaque, 350 mg L/mL, GE Healthcare, Oslo, Norway). Images were acquired in a soft tissue reconstruction algorithm 45 s after the contrast medium. CT of the sheep’s head was taken in axial view, and a multiplanar reconstruction was obtained in the image post-processing stage. CT confirmed the presence of a large intra-axial cystic formation located predominantly within the left cerebral hemisphere. The lesion had a homogeneous hypoattenuating content of approximately 6.2 Hounsfield units, iso-attenuating relative to the cerebrospinal fluid, and a discretely bilobate configuration. It measured approximately 3.4 × 3.0 × 3.6 cm in the ventrodorsal, mediolateral, and craniocaudal planes, respectively. The cyst walls were well delimited but of variable thickness. Dorsally, the wall was discretely irregular, relatively hyperattenuating and of fine granular appearance—an aspect consistent with aggregates of protoscolices. Ventrally, it was thin and poorly delineated; a few incomplete septa were present cranio-dorsally (Figure 1 and Figure 2).

The formation extended caudally into the caudal cranial fossa, without evident involvement of the cerebellar parenchyma, and exerted a severe mass effect, with displacement and compression of the adjacent intracranial structures. The ventricular system was markedly deformed and displaced, the thalamic structures were compressed, and the median line was deviated contralaterally. The granular mural structures were most evident along the dorsal aspect of the lesion. A parasitic cyst of hydatid type, an arachnoid or porencephalic cyst and—less probably—a chronic cerebral abscess were considered as differential diagnoses; the cerebral localization, the almost fluid content, the large size, the partly granular wall, and the presence of septa were regarded as characteristic of a parasitic cyst, with cerebral coenurosis being the principal diagnosis in sheep.

The severe mass effect described above accounts for the potential prosencephalic and diencephalic neurological signs, the caudal extension being present without evident cerebellar invasion.

The electroencephalographic recording was obtained after the computed tomographic examination and after euthanasia had already been elected by the owner on prognostic grounds, as detailed below. EEG was performed under sedation with ketamine (Ketamidor 10%, VetViva Richter GmbH, Wels, Austria) at 10 mg/kg by intravenous injection, in association with diazepam (Diazepam 5 mg/mL, Terapia, Cluj-Napoca, Romania) at 1 mg/kg by intravenous injection, using a Neurofax electroencephalograph (Nihon Kohden, Tokyo, Japan). Recording electrodes (subcutaneous electrodes with 0.22 × 22.5 mm connector and 1.5 mm needle (stainless steel) (NE-224S EEG, Nihon Kohden, Tokyo, Japan)) were positioned according to a reduced montage derived from the human 10–20 system (F3, C3, O1, F4, C4, O2, referenced to the ipsilateral auricular electrodes A1/A2), complemented by longitudinal and transverse bipolar derivations. Signals were digitized at 500 Hz (sampling interval 2 ms). Two consecutive electroencephalographic recordings were obtained from the same animal, rather than a single tracing, for methodological reasons. Firstly, the EEG acquired under sedation is strongly modulated by the anesthetic agent and by the depth and phase of sedation, with distinct agents and sedation levels producing characteristic and non-interchangeable spectral signatures [11,12]; a single recording therefore samples only one anesthetic state, whereas two recordings separated in time provide a within-subject gradient of sedation against which the stability of each quantitative feature can be judged. Secondly, repeated recording allows state-dependent features, which vary with the anesthetic plane, to be distinguished from trait-like features, which persist across recordings and are more plausibly related to the underlying disease; this distinction is particularly relevant for the slow-frequency measures, whose test–retest reliability is known to be lower than that of the faster rhythms [13]. Thirdly, and as is required in human clinical neurophysiology before a quantitative electroencephalographic marker is accepted as reliable [13,14], reproducibility across sessions was sought as evidence of robustness; the acquisition of two tracings additionally provided a within-subject replication that increased the amount of artefact-free data available for spectral estimation and reduced the risk of basing the analysis on a single, potentially unrepresentative recording, thereby maximizing the information obtained from one animal.

For quantitative analysis (qEEG), the six referential derivations were segmented into 4-s epochs with 50% overlap and analyzed by Welch power spectral density estimation (frequency resolution 0.25 Hz). Epochs were excluded from analysis if any derivation reached the acquisition rail, if any derivation was flat, or if the epoch amplitude was an extreme outlier (robust z-score above 5, computed on the log standard deviation across derivations); 867 of 901 epochs (96.2%) were retained in the first recording and 637 of 683 (93.3%) in the second. Absolute and relative band powers were computed for delta (0.5–4 Hz), theta (4–8 Hz), alpha (8–12 Hz), beta (12–30 Hz), and gamma (30–45 Hz). Median frequency, the 95% spectral edge frequency (SEF95), the delta/alpha ratio (DAR), and the (delta + theta)/(alpha + beta) ratio were derived per derivation. Interhemispheric asymmetry was expressed as an asymmetry index, AI = (L − R)/(L + R), computed per homologous electrode pair and frequency band from the absolute band powers of each retained epoch. Because a single point estimate of AI conveys no information about its precision, the epoch-wise distribution was used to derive a 95% confidence interval by non-parametric bootstrap resampling of epochs (10,000 resamples, percentile method). An asymmetry was considered reproducible only if the confidence intervals of both recordings excluded zero and the sign of the index was concordant between recordings; asymmetries whose interval included zero in either recording, or whose sign reversed between recordings, were treated as non-reproducible. Mean absolute band powers were additionally compared between homologous derivations in order to establish whether any asymmetry arose from attenuation over one hemisphere or from augmentation over the other. The burst suppression ratio (BSR) was quantified from the amplitude envelope of the averaged central derivations. Analyses were performed in Python 3.12.3 (NumPy 2.4.4, SciPy 1.17.1, pandas 3.0.2). 

### 2.1. Visual EEG

Both recordings showed a continuous background, without discontinuity or a burst suppression pattern (less than 0.2% of the tracing was suppressed). The background was dominated by high- to moderate-voltage slow (delta) activity with a dominant frequency of 1.25–3.75 Hz, admixed with lower-voltage faster (beta) rhythms, an appearance compatible with ketamine–diazepam sedation [12,15]. Interhemispheric symmetry was grossly preserved on visual inspection, with only a subtle excess of slow activity over the right hemisphere. No rhythmic epileptiform discharges and no electrographic seizures were identified; occasional isolated sharp transients were of uncertain significance. Standardized reactivity testing was not recorded (the trigger channel contained no event markers). The second recording, obtained approximately 48 min later, was of lower voltage and richer in fast activity, in keeping with a lighter plane of sedation.

### 2.2. Quantitative EEG

The quantitative indices are summarized in Table 1. In both recordings, the spectrum was delta-dominant (relative delta power 42.4% and 43.2%, respectively), with a delta/alpha ratio well above unity (3.65 and 4.02) and a low median frequency (4.50 and 4.67 Hz). From the first to the second recording, the composition shifted towards faster, lower-voltage activity: relative beta power increased (14.7% to 18.8%), SEF95 increased (20.9 to 25.0 Hz), relative theta power decreased (24.5% to 19.8%), the (delta + theta)/(alpha + beta) ratio decreased (2.59 to 2.24), and the mean amplitude decreased (13.3 to 10.8 µV). The burst suppression ratio remained negligible in both recordings (0.05% and 0.64%).

**Figure 3:**
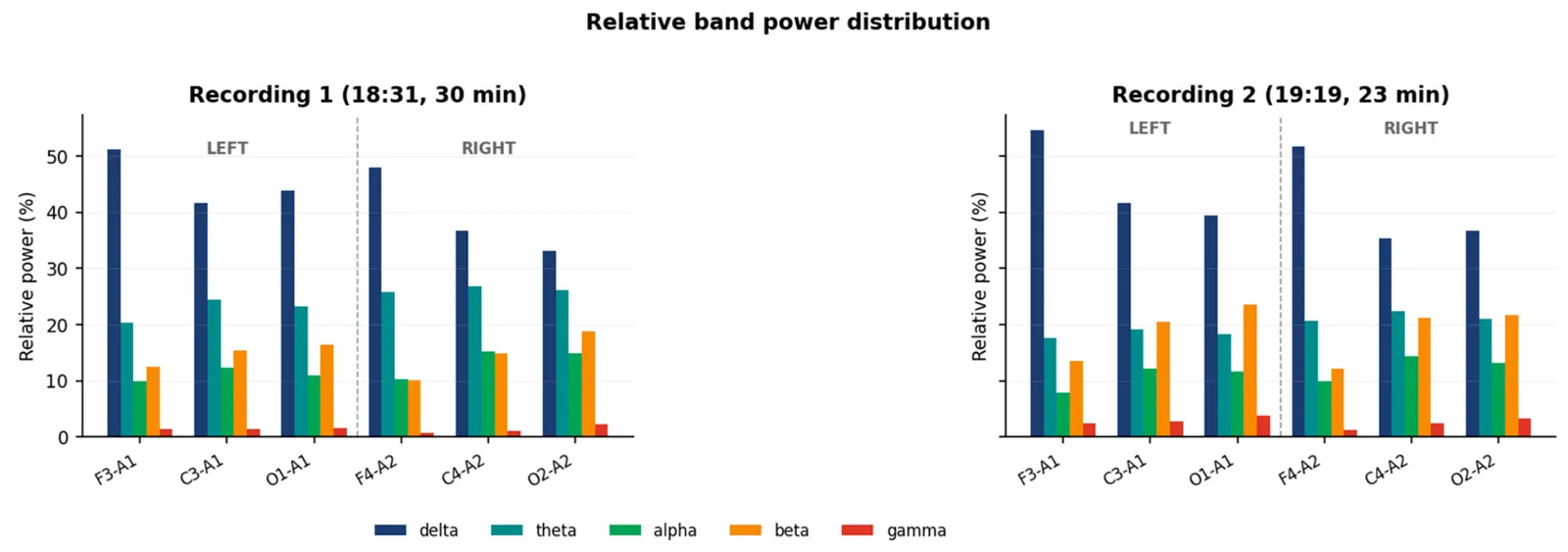
Relative band-power distribution across the six referential derivations for the two recordings; left- and right-hemisphere derivations are grouped. Note the delta-dominant composition and the posterior increase in beta power in the second recording.

**Figure 4:**
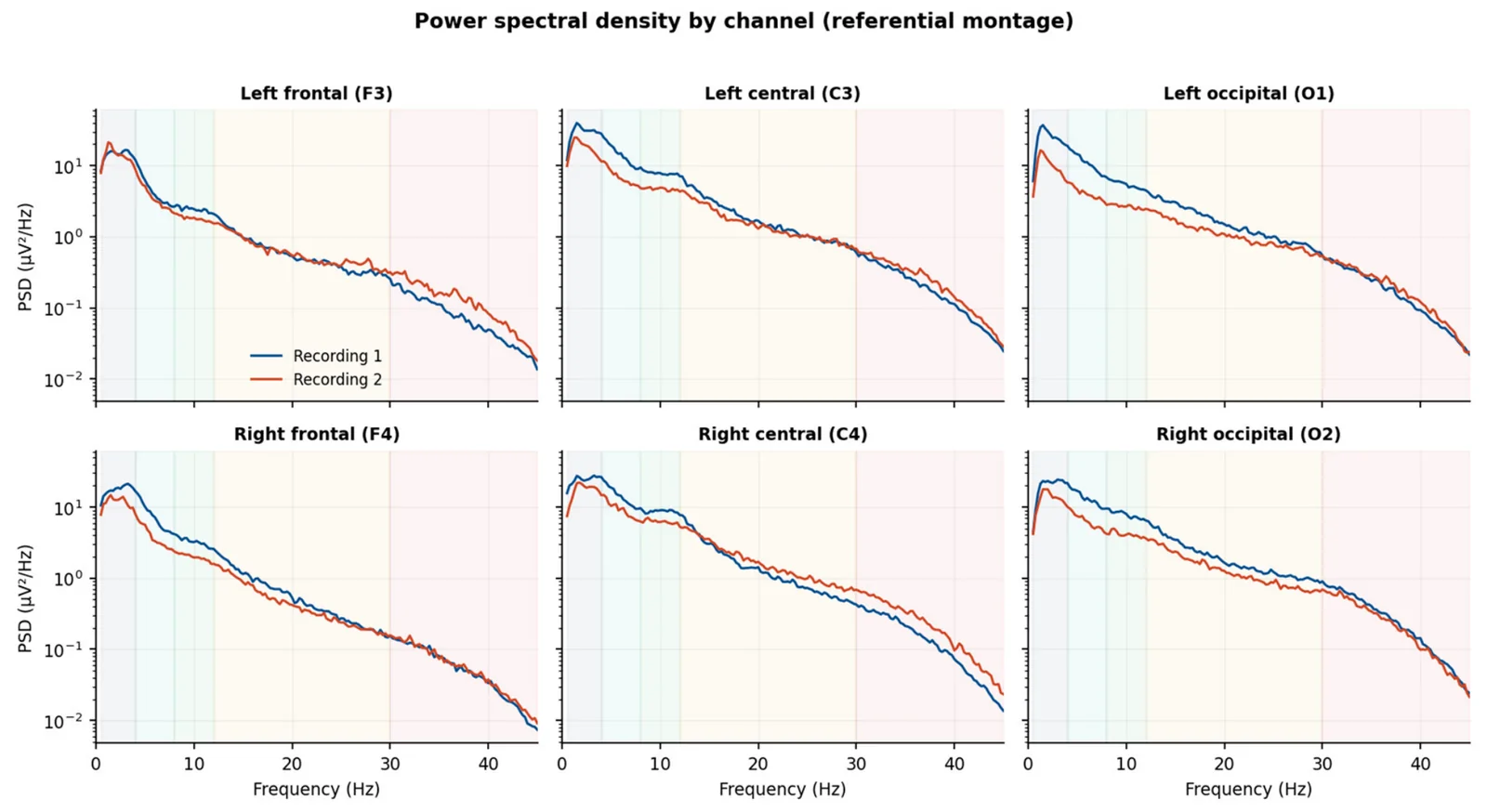
Channel-wise power spectral density (referential montage). Shaded bands denote the classical frequency ranges; the spectra decline steeply from the delta range and are broadly comparable between hemispheres.

The power spectral density profiles and their regional distribution are shown in Figure 3 and Figure 4.

**Figure 5:**
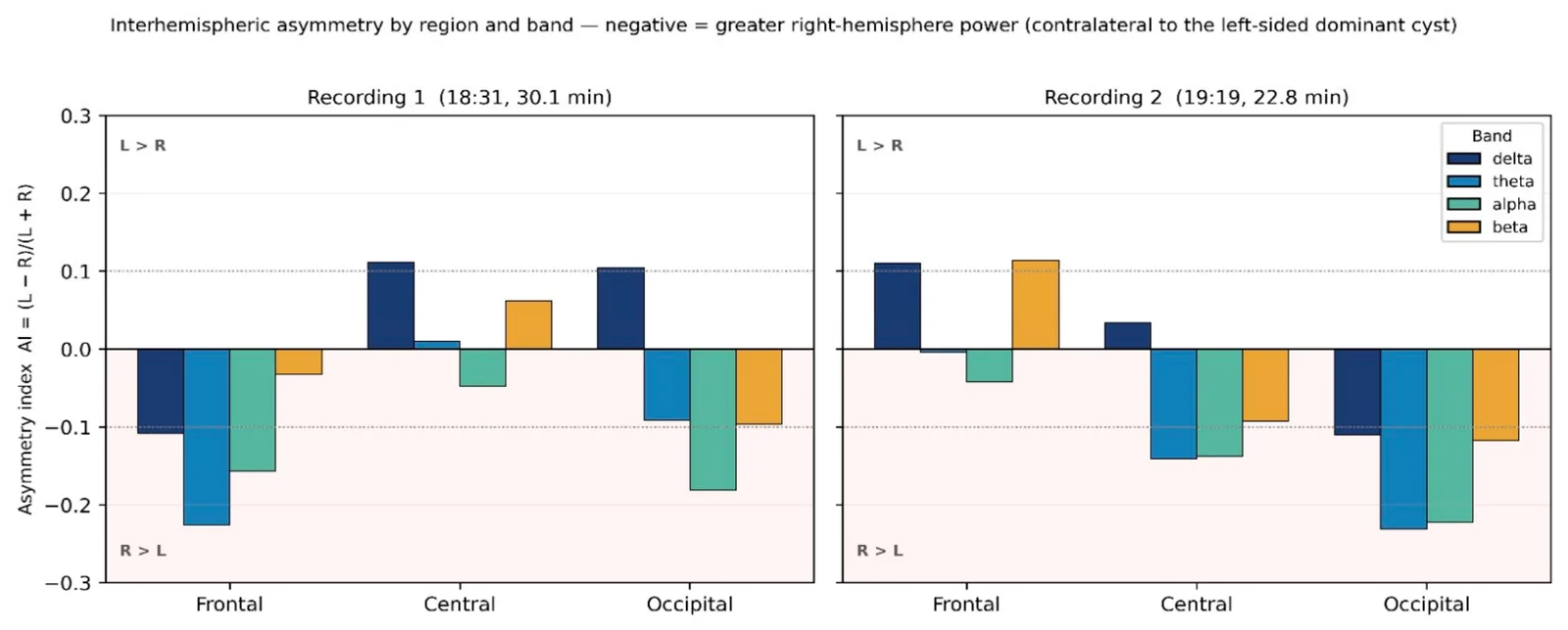
Interhemispheric asymmetry of spectral power by region and band for the two recordings. The asymmetry index (AI = (L − R)/(L + R)) is plotted so that positive values indicate greater left-hemisphere power and negative values greater right-hemisphere power; dotted lines mark ±0.10, and the shaded lower half marks the right-hemisphere (negative) domain, contralateral to the left-sided cyst. The frontal and central indices are unstable, several reversing in sign between sessions, whereas the occipital indices lie over the right hemisphere in both recordings in every band except delta. The reproducible component of the asymmetry is therefore occipital, right-sided, and discordant with the side of the structural lesion.

Interhemispheric asymmetry indices, with their bootstrap confidence intervals, are given in Table 2 and Figure 5. Because several hundred epochs contributed to each estimate, the confidence intervals were narrow, and most indices differed significantly from zero within a given recording; precision within a recording, however, is not the same as reproducibility across recordings, and the two were assessed separately. Of the eighteen region–band combinations examined, nine met the criterion for reproducibility. These were not distributed at random. All five of the reproducible occipital indices lay over the right hemisphere and were concordant in sign across both recordings: theta (AI −0.094 and −0.224), alpha (−0.180 and −0.205), beta (−0.100 and −0.129), gamma (−0.185 and −0.101), and total power (−0.036 and −0.157). By contrast, the frontal and central asymmetries were unstable: frontal delta, beta and total power and central theta, beta, and total power reversed in sign between the two recordings, and occipital delta likewise reversed (+0.109 to −0.090). The reproducible right-sided preponderance was therefore confined to the occipital derivations and was present in every band except delta.

**Figure 6:**
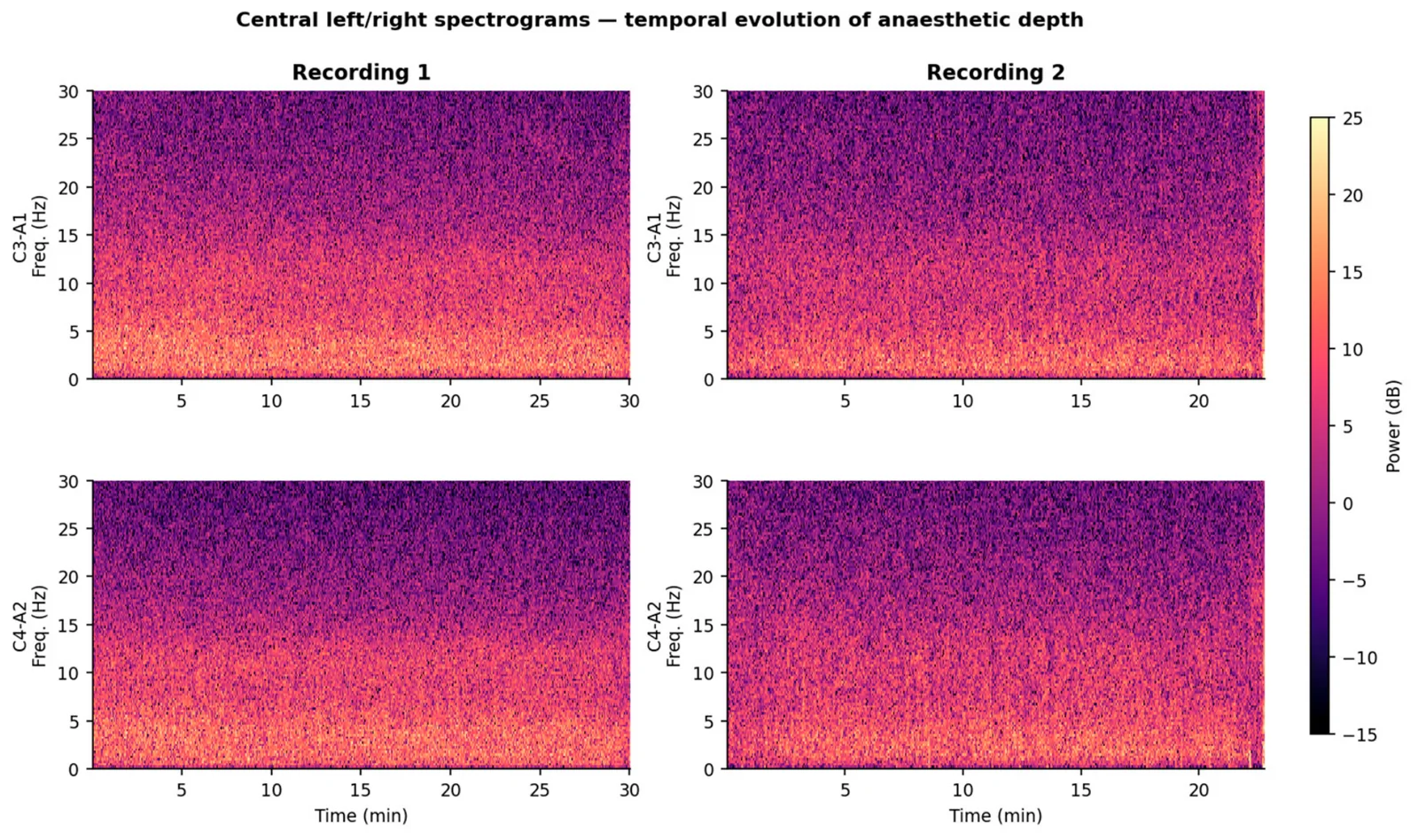
Central left/right spectrograms for both recordings, illustrating the sustained low-frequency dominance and the temporal evolution of the sedation plane.

Comparison of the absolute band powers indicated that this preponderance arose principally from lower power recorded over the left hemisphere rather than from higher power over the right. Total absolute power averaged across the three left-sided derivations was 0.96 and 0.88 times that of the homologous right-sided derivations in the first and second recording, respectively, and the difference was most marked at the occipital pair, where the O1/O2 ratios were 0.94 and 0.72. The lower voltage was thus recorded over the hemisphere containing the cyst. The sustained predominance of low-frequency activity throughout both recordings, and the gradual shift towards faster activity in the second, are also apparent in the time–frequency representation of the central derivations (Figure 6).

Owing to the extent of the lesion and to the poor prognosis established by the neurological examination and by CT, the owner elected euthanasia of the animal before the electroencephalographic recordings were obtained and consented to the necropsy and subsequent histopathological examination for scientific purposes. The electroencephalographic recording was therefore obtained under sedation, immediately prior to euthanasia, using fine subcutaneous needle electrodes; it did not contribute to the clinical decision, did not alter the clinical course, and did not prolong the life or the suffering of the animal.

**Figure 7:**
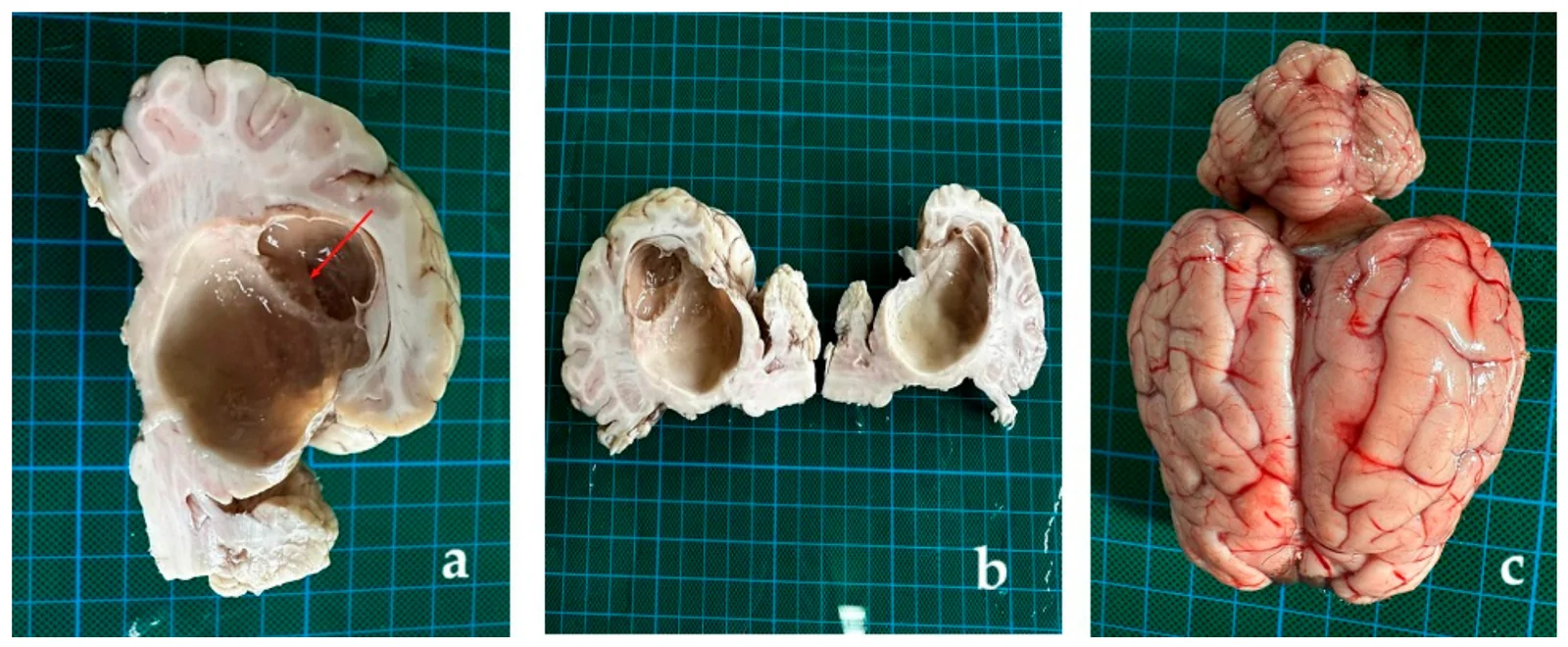
Solitary cystic parasitic granuloma in the left cerebral hemisphere of the sheep. (**a**,**b**) Longitudinal sections showing the bilobate cyst and compression atrophy of the adjacent nervous tissue; red arrow—protoscolices. (**c**) Dorsal aspect of the intact brain, showing the caudal extension of the lesion with displacement and compression of the brainstem.

After brain extraction and longitudinal sectioning of both cerebral hemispheres, a single large cystic parasitic granuloma was identified within the left cerebral hemisphere, in a parieto-occipital position, of discretely bilobate configuration and containing incomplete septa, corresponding to the appearance demonstrated on computed tomography; no cyst and no parasitic granuloma were found in the right cerebral hemisphere (Figure 7a,b). On the sectioned specimen, the lesion measured approximately 5 cm in its greatest anteroposterior extent. Postmortem and in vivo dimensions are not directly comparable, as the removal of the brain from the cranial vault releases the constraint imposed by the intact calvarium and by the raised intracranial pressure, and the plane of the section does not necessarily correspond to that of the tomographic measurement. Caudally, the cyst extended towards the caudal cranial fossa and compressed and displaced the brainstem, as acknowledged upon dorsal inspection of the intact brain (Figure 7c), without macroscopic evidence of a discrete parasitic lesion within the brainstem itself or of cerebellar invasion.

It appeared as a cyst with a thin, almost transparent membrane, filled with a clear, transparent liquid and with fine whitish formations attached to the internal surface (protoscolices—red arrow). The cyst produced a significant compression atrophy of the bordering nervous substance and—caudally—a marked compression of the brainstem.

**Figure 8:**
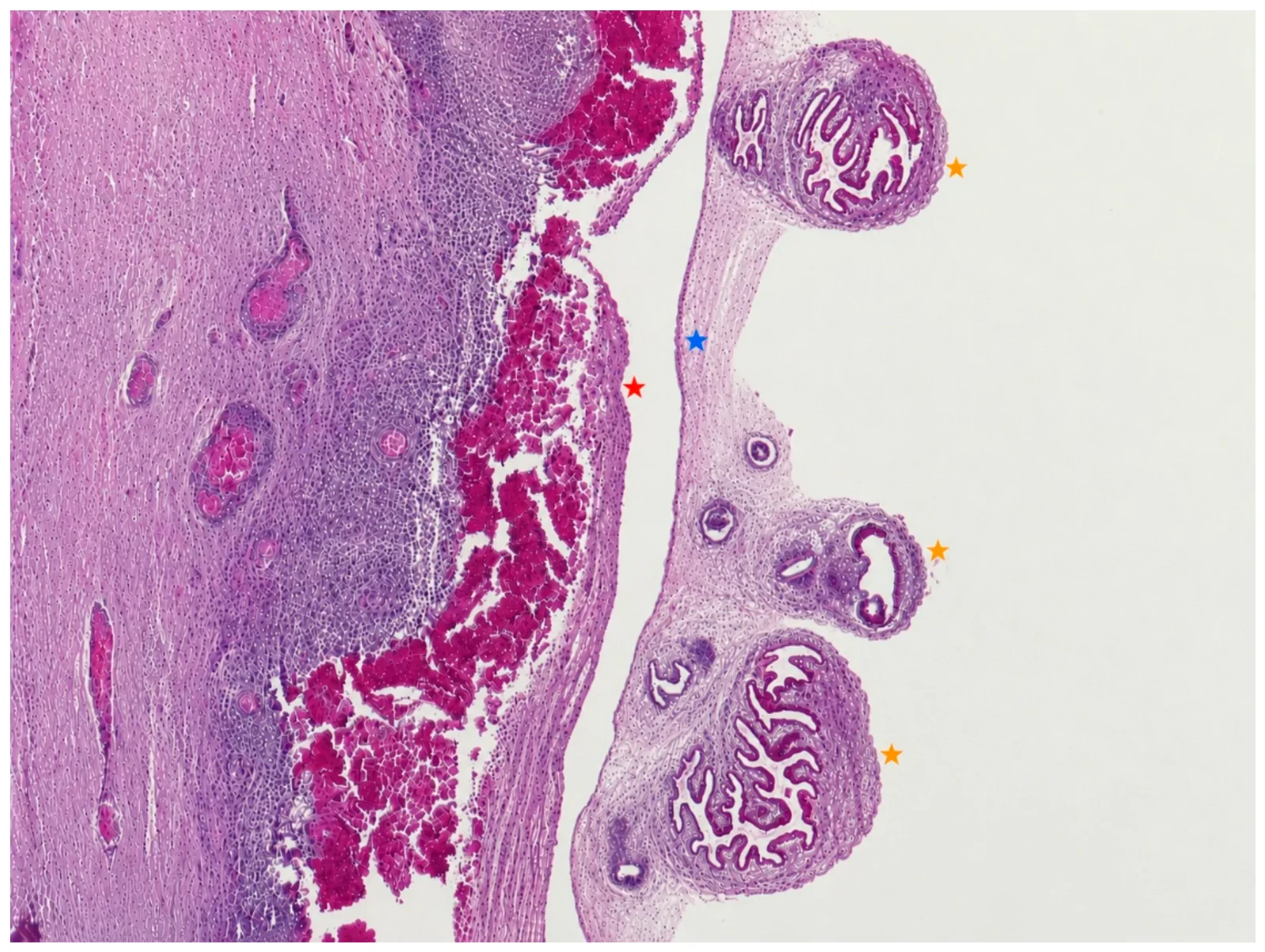
Histopathological aspect of the cerebral lesion produced by the infestation with *Coenurus cerebralis*, col. Trichromic Masson. red star—perivascular cuffing with lymphocytes/macrophages blue star—cyst’s capsule orange star—protoscolices.

Histopathological examination of the brain revealed a characteristic *Coenurus cerebralis* parasitic granuloma, showing compression atrophy, neuronal damage, inflammation (gliosis, perivascular cuffing with lymphocytes/macrophages, and eosinophils—red star), and cystic structures with invaginated protoscolices (head-like structures with suckers—orange star) surrounded by a capsule of fibrous tissue and giant cells (blue star), leading to neurological signs or tissue lesions (Figure 8).

## 3. Discussion

To our knowledge, this is the first description of the electroencephalographic (EEG) and quantitative electroencephalographic (qEEG) features of ovine coenurosis correlated with computed tomography (CT). This study demonstrated that a sheep with a solitary left-sided brain cyst (coenurosis confirmed by CT and at necropsy), sedated with ketamine and diazepam, presented an EEG pattern characterized by continuous background activity dominated by delta waves. Superimposed on this pattern were a mild and unstable interhemispheric amplitude asymmetry and isolated sharp waves on the right side (that is, over the hemisphere contralateral to the only demonstrable structural lesion). Whereas the parasitology, pathology, immunology and cross-sectional imaging of coenurosis are well documented [1,3,7], its electrophysiological phenotype has not previously been characterized.

Any interpretation of these recordings must begin with the electroencephalographic signature of the anesthetic protocol, since the background rhythm of a sedated animal is determined more by the drugs administered than by any focal pathology. The cortical EEG response of the sheep to ketamine has been characterized directly: ketamine induces an immediate, spectrally widespread change and, during the sedated phase, a background dominated by low-frequency (delta) activity before giving way in recovery to a phase of alternating low- (<14 Hz) and high- (>35 Hz) frequency oscillation that is thought to underlie the dissociative state; these effects are dose-dependent, and at high intravenous doses (24 mg/kg), cortical activity may cease entirely, producing an isoelectric “k-hole” state [16]. In quantitative terms, the present recordings were delta-dominant on every derivation (relative delta approximately 42%, versus approximately 17% beta), a composition that corresponds to this sedated ovine phase rather than to an alert, beta-predominant pattern; the isoelectric “k-hole” was not observed, consistent with the moderate dose employed (10 mg/kg), well below the threshold reported in the sheep. The superimposed beta component is, in turn, the expected contribution of the co-administered diazepam, benzodiazepines being among the most consistent “beta activators” of the cortical EEG [11,15]. It is therefore likely that the slow-wave predominance and the superimposed fast activity were generated principally by the ketamine–diazepam combination, and that the shift towards faster, lower-voltage activity in the second recording reflects a time- and dose-dependent lightening of sedation—a transition towards the dissociative, alternating ovine pattern—rather than any change in the underlying disease.

In human clinical neurophysiology, slow-wave activity is a recognized correlate of neoplastic, ischemic and demyelinating brain lesions, its abundance and amplitude depending on the degree of cortical involvement and on the depth of the lesion relative to the recording electrodes, the classic sign being continuous focal polymorphic delta activity attributed to white-matter involvement with functional deafferentation of the overlying cortex [17,18]. In the present animal, the cyst was periventricular, with only indirect cortical involvement through perilesional oedema and compression atrophy secondary to mass effect. Such a configuration is expected to produce diffuse or mildly regional slowing rather than a sharply localized polymorphic delta focus, and this expectation accords with the observed pattern.

Against this pharmacological background, the lesional contribution to the interhemispheric asymmetry was examined on the referential montage, as recommended for amplitude comparisons. Contrary to the expectation that the dominant slow-band excess would lie ipsilateral to the structural lesion, the asymmetry indices did not lateralize towards the CT-confirmed left-sided cyst. The addition of confidence intervals allowed a distinction that a single point estimate had obscured to be drawn. Part of the asymmetry was demonstrably unstable: frontal delta, beta and total power, central theta, beta and total power, and occipital delta all reversed in hemispheric sign between the two recordings. These results are most plausibly attributable to the changing plane of sedation rather than to the lesion. A second component, however, was reproducible. In the occipital derivations, the asymmetry lay over the right hemisphere in both recordings in theta, alpha, beta, gamma and total power, with confidence intervals excluding zero on every occasion. The interpretation offered below therefore applies to this reproducible occipital component alone and not to the unstable frontal and central indices. Since neither computed tomography nor necropsy demonstrated any lesion within the right cerebral hemisphere, this reproducible right-sided preponderance cannot be attributed to a structural generator on that side, and an indirect mechanism has to be sought.

Two mechanisms may account for a reproducible asymmetry directed away from the structural lesion, and the present data allow them to be ranked. In human clinical neurophysiology, a lateralized polymorphic delta focus is characteristically produced by superficial, cortically based lesions with white-matter involvement, whereas deep, periventricular generators are poorly sampled by surface electrodes and tend to project diffusely or even to the contralateral hemisphere through volume conduction and interhemispheric network effects [17,18]. In the present animal, the cyst was deep and periventricular, with only indirect cortical involvement through perilesional compression atrophy, a configuration that would be expected to degrade rather than sharpen surface lateralization. The first mechanism is attenuation of the signal recorded over the diseased hemisphere. A large cyst whose contents are iso-attenuating with cerebrospinal fluid interposes a highly conductive compartment between the cortical generators and the recording electrodes, and the perilesional compression atrophy demonstrated histologically further reduces the volume of functioning cortex beneath the left derivations. Both effects lower the voltage recorded over the affected side and, because the asymmetry index is a normalized quantity, displace it towards the healthy hemisphere without any change in the latter. The absolute powers support this interpretation: total power over the left derivations was 0.96 and 0.88 times that over the right, and the ratio fell to 0.94 and 0.72 at the occipital pair, so that the reduction in voltage was located over the hemisphere containing the cyst. Attenuation over a damaged hemisphere is the mechanism most often invoked to explain false lateralization of the surface electroencephalogram in human patients. The second mechanism is an active contribution from the contralateral hemisphere, which was itself displaced and compressed: the cyst deformed and displaced the ventricular system, compressed the thalamic structures, and deviated the median line contralaterally. A directly comparable observation has been reported in human patients, in whom focal lesions producing midline shift were accompanied by lateralized periodic discharges and seizures arising from the hemisphere contralateral to the lesion [19]; this phenomenon belongs to the wider family of false localizing signs, among which the compression of the contralateral cerebral peduncle against the tentorial edge described by Kernohan and Woltman is the classical instance [20]. The two mechanisms are not mutually exclusive, and the present recordings cannot separate them, as no normative ovine data and no contemporaneous control recording were available. On the evidence of the absolute powers, however, attenuation over the diseased hemisphere is the more parsimonious of the two. Whichever predominates, the practical consequence is the same, and this is the point of clinical interest: the side of the surface electroencephalographic abnormality did not indicate the side of the lesion. The right-directed head tilt and circling and the right-sided brainstem deficits are attributable to a related but anatomically distinct consequence of the same expanding mass, namely the caudal displacement and compression of the brainstem demonstrated at necropsy, so that a single pathological process may be seen to act at two levels rather than through one mechanism. The coarse six-electrode montage and the anesthesia-imposed slowing may reasonably be presumed to have compounded this effect.

Isolated sharp transients (70–200 ms, surface-negative) were observed on the right central and occipital derivations, maximal at the C4 electrode and without contralateral spread. Their interpretation requires caution. Ketamine has recognized proconvulsant properties and can itself generate sharp cortical activity; the acquisition range was clipped; and high-amplitude frontal transients are, in this species, most often of ocular origin—all of which must be excluded before a transient is regarded as epileptiform. Notably, these right-sided transients lay contralateral to the solitary left-sided cyst, reinforcing the impression that the surface EEG did not track the side of the principal structural lesion; a deep periventricular lesion is, moreover, classically associated with bilateral, symmetrical discharges and an unchanged background rhythm, rather than with a unilateral superficial focus [12,16]. With these disclaimers in mind, and if genuine epileptiform morphology is confirmed on visual review, the right centro-occipital transients would at most indicate a focal cortical irritative zone remote from the cyst itself; their topography, over the same derivations that carried the reproducible right-sided spectral asymmetry, is at least consistent with the mechanisms proposed above. In the absence of clinically or electrographically documented seizures, however, the attribution of a discrete epileptogenic focus remains to be confirmed.

These observations align with the wider literature on cystic and space-occupying brain lesions across species. Among the human parasitic cysts, neurocysticercosis is the closest analogue, sharing a cestode etiology (*Taenia solium*) and a histological appearance similar to that of *Coenurus cerebralis* [21]. In a series of 203 patients with epilepsy attributable to neurocysticercosis, focal electroencephalographic abnormalities were present only in those harboring a single cyst and were absent in patients with multiple cysts [22]. The present animal harbored a single cyst and nevertheless showed no focal abnormality ipsilateral to it. This comparison is not offered as evidence of a difference between species, which a single animal set against a cohort of that size could not establish; the human series did not select for lesions producing severe mass effect with midline displacement, and we offer such displacement as one hypothesis consistent with the present single observation rather than as an established determinant. Human cerebral coenurosis, a recognized zoonosis, produces raised intracranial pressure, seizures, and focal deficits, including the trigeminal and facial involvement seen here [1,23,24]. In veterinary patients, eight dogs with intracranial mass lesions showed slow-wave activity in most animals but an amplitude or frequency asymmetry in only about half, with no pathognomonic pattern [25]. Among the compressive and cystic canine conditions, hydrocephalus is associated with slowing, epileptiform discharges, and interhemispheric asynchrony [25], whereas quadrigeminal cysts lack a specific EEG signature and, when abnormal, most often show interictal epileptiform discharges in a minority of cases [8]. The present findings—diffuse slowing with a reproducible occipital asymmetry contralateral to the cyst—align with this collective picture, in which cystic space-occupying lesions produce inconsistent and only weakly lateralizing surface EEG changes.

Beyond its electrophysiological interest, the present case carries a One Health dimension that deserves emphasis. *Taenia multiceps* is a zoonotic cestode: the dog, as definitive host, sheds gravid proglottids onto pasture and perpetuates the cycle in sheep, whereas humans may act as accidental intermediate hosts and develop cerebral coenurosis, with raised intracranial pressure, seizures, and focal deficits [19,21]. Control therefore rests on measures that interrupt transmission to both species, namely the restriction and regular anthelmintic treatment of farm dogs and the safe disposal of infected ovine offal rather than dog-accessible discarding [1]. In addition, ovine coenurosis offers a naturally occurring large-animal counterpart of human cerebral coenurosis and neurocysticercosis, in which the dimensions of the ovine cranium permit imaging and neurophysiological protocols closer to those of human medicine than are feasible in rodent models; the present characterization is offered in that translational spirit.

When compared with the awake human, in whom a hemispheric mass typically yields focal continuous polymorphic delta activity, the sedated ovine coenurotic electroencephalogram displayed a different phenotype, i.e., diffuse anesthesia-shaped slowing upon which a reproducible occipital asymmetry, directed away from the imaging lesion, and isolated sharp transients were superimposed [17,20]. The principal contributions of this report are therefore two: this report provides the first visual and quantitative EEG characterization of ovine coenurosis, and it demonstrates that the side of the surface electroencephalographic abnormality need not correspond to the side of the structural lesion when that lesion produces a severe mass effect. The recording was not uninformative, since it reflected a genuine and reproducible functional asymmetry; what it did not do was indicate where the lesion lay. Electroencephalographic lateralization should accordingly be read, in such circumstances, as a potential false localizing sign rather than as a direct indicator of lesion side. The phenomenon is not itself new—it is documented in human patients [23,24]—but it has not previously been described in a ruminant, nor in coenurosis. In doing so, it positions ovine coenurosis as an informative large-animal counterpart of human parasitic space-occupying brain disease and is consistent with the established use of the sheep as a model for the cerebral actions of ketamine. Further studies in larger numbers of animals, ideally with recordings obtained in the unsedated or lightly sedated state and correlated with advanced imaging, are required to determine whether focal qEEG slowing and sharp transients can serve as reliable lateralizing markers in ovine coenurosis.

The value of acquiring two recordings is well illustrated by the present findings. The delta-dominant background and the low median frequency were reproducible across both tracings and are therefore likely to represent a stable, state-related feature of the sedated animal, whereas the interhemispheric asymmetry was not reproducible, differing in both magnitude and hemispheric direction between the two recordings. This dissociation could not have been recognized from a single recording, in which an incidental slow-band asymmetry might have been over-interpreted as a stable, lesion-related lateralizing sign. Furthermore, the shift towards faster, lower-voltage activity in the second recording is consistent with a lightening of the sedation plane and with the known time- and dose-dependent electroencephalographic dynamics of ketamine, which produce alternating slow and fast activity together with increased theta and reduced alpha–beta power [11,12]. In our case, the clinical examination and the CT lateralized the lesion to the left (whereas the surface qEEG under ketamine–diazepam sedation did not) and should be regarded as a complement to—rather than a substitute for—cross-sectional imaging in the neurolocalization of ovine coenurosis.

Although the present study characterized the neurological and electroencephalographic phenotype of this case with imaging correlation, several limitations should be considered. Firstly, the montage comprised only six active electrodes derived from the human 10–20 system and applied to the ovine cranium, so the spatial resolution was coarse and the electrode-to-cortex correspondence uncertain; the referential derivations were nonetheless analyzed consistently to limit this effect. The constraints imposed by the ruminant head compound this limitation, as the thick cranial vault, the extensive temporalis musculature, and the particular skull morphology of small ruminants increase the susceptibility to muscle artefact and to volume conduction, and deep periventricular generators are intrinsically poorly resolved by coarse surface derivations. Future work should therefore employ higher-density surface montages or epidural electrode placement, ideally in unsedated or minimally sedated animals. The gamma-band indices should be regarded with particular reserve, since activity above 30 Hz recorded over the ovine head is readily contaminated by temporalis electromyographic activity. Secondly, the EEG was acquired under ketamine–diazepam sedation, which imposes its own slow-wave signature and, through the proconvulsant action of ketamine, complicates the interpretation of sharp transients; the absence of a burst suppression or “k-hole” pattern indicates a moderate rather than deep plane, but reactivity was not formally tested. It must be conceded that the diffuse high-amplitude delta background imposed by this protocol may mask focal lesional slowing, so that the absence of a lateralizing slow-wave focus cannot be attributed with confidence to an intrinsic property of *Coenurus cerebralis* lesions and may in part be pharmacological in origin. Thirdly, the two recordings differed in acquisition range (the signal was clipped at ±100 µV and ±70 µV, respectively, affecting 0.05% and 0.20% of samples; epochs containing such samples were excluded from the quantitative analysis), so that absolute amplitudes are not directly comparable between sessions, and relative power was used for between-recording comparison. Fourthly, the interpretation advanced here rests on a distinction between attenuation over the diseased hemisphere and augmentation over the compressed contralateral hemisphere that the present data cannot resolve, since no normative ovine values and no contemporaneous control recording were available; the supporting evidence is limited to the direction of the absolute power difference. Fifthly, the deviation of the median line was reported qualitatively by the examining radiologist and was not quantified in millimeters. Thus, the magnitude of the displacement cannot be related to that of the electrophysiological asymmetry, as would be desirable; retrospective measurement was no longer possible. Furthermore, the absence of a focal abnormality ipsilateral to the cyst may reflect normal biological variability or individual variation in the characteristics of the lesion, rather than the mass-effect mechanism proposed here, and a single case cannot distinguish between these possibilities. Lastly, the observations derive from a single animal, so that the mechanisms proposed to explain the contralateral electrophysiological preponderance remain an interpretation of one case and require confirmation in a larger series.

## 4. Conclusions

In summary, the EEG of a sheep harboring a CT- and necropsy-confirmed solitary left-sided coenurotic cerebral cyst was dominated, under ketamine–diazepam sedation, by a continuous slow-wave (delta) background. Quantitative analysis with bootstrap confidence intervals separated an unstable asymmetry—which reversed sides between recordings and is attributable to the changing plane of sedation—from a reproducible occipital asymmetry directed over the right hemisphere, in which no structural lesion existed. Absolute powers indicated that this reproducible asymmetry arose chiefly from attenuation over the diseased hemisphere. The side of the surface qEEG abnormality therefore did not indicate the side of the structural lesion identified on CT.

The application of EEG could nonetheless provide valuable insights into the functional brain disturbances caused by *Taenia multiceps* metacestodes and may offer a non-invasive complementary tool, particularly for the assessment of cyst-related cortical dysfunction. Its value for neurolocalization in ovine coenurosis, however, remains to be established: the present observation suggests that, in the presence of a severe mass effect, electrophysiological lateralization may constitute a false localizing sign and should not be read as a direct indicator of the side of the lesion. Confirmation is required in a larger series, ideally with recordings obtained in the unsedated or lightly sedated state and correlated with advanced imaging.

To our knowledge, this represents the first detailed descriptions of qEEG findings in ovine coenurosis, providing new insights into the neurophysiological consequences of cerebral parasitic cysts in sheep.

## Figures and Tables

**Table 1:** Quantitative EEG indices, averaged across the six referential derivations, for the two recordings.

Parameter	Recording 1 (30.1 min)	Recording 2 (22.8 min)
Relative delta power (%)	42.4	43.2
Relative theta power (%)	24.5	19.8
Relative alpha power (%)	12.2	11.5
Relative beta power (%)	14.7	18.8
Relative gamma power (%)	1.4	2.7
Median frequency (Hz)	4.50	4.67
Spectral edge frequency 95% (Hz)	20.9	25.0
Delta/alpha ratio (DAR)	3.65	4.02
(Delta + theta)/(alpha + beta) ratio	2.59	2.24
Mean amplitude, SD (µV)	13.3	10.8
Burst suppression ratio (%)	0.05	0.64

**Table 2:** Interhemispheric asymmetry index, AI = (L − R)/(L + R), by region, frequency band and recording, with 95% bootstrap confidence intervals (10,000 resamples over epochs). Negative values indicate greater right-hemisphere power. An index was classified as reproducible only if both confidence intervals excluded zero and the sign was concordant between recordings.

Region	Band	AI (Rec. 1) [95% CI]	AI (Rec. 2) [95% CI]	Reproducible
Frontal	Delta	−0.140 [−0.152, −0.128]	+0.130 [+0.115, +0.145]	No—sign reversal
Frontal	Theta	−0.228 [−0.242, −0.215]	−0.015 [−0.031, +0.001]	No—CI includes 0
Frontal	Alpha	−0.159 [−0.173, −0.146]	−0.038 [−0.054, −0.022]	Yes
Frontal	Beta	−0.039 [−0.047, −0.030]	+0.100 [+0.090, +0.110]	No—sign reversal
Frontal	Gamma	+0.144 [+0.133, +0.156]	+0.304 [+0.289, +0.318]	Yes
Frontal	Total	−0.151 [−0.159, −0.144]	+0.081 [+0.071, +0.090]	No—sign reversal
Central	Delta	+0.111 [+0.100, +0.121]	+0.038 [+0.022, +0.055]	Yes
Central	Theta	+0.004 [−0.007, +0.015]	−0.140 [−0.155, −0.125]	No—sign reversal
Central	Alpha	−0.049 [−0.059, −0.039]	−0.129 [−0.143, −0.115]	Yes
Central	Beta	+0.059 [+0.052, +0.067]	−0.093 [−0.102, −0.083]	No—sign reversal
Central	Gamma	+0.148 [+0.137, +0.159]	+0.008 [−0.007, +0.024]	No—CI includes 0
Central	Total	+0.050 [+0.044, +0.056]	−0.059 [−0.067, −0.051]	No—sign reversal
Occipital	Delta	+0.109 [+0.096, +0.122]	−0.090 [−0.107, −0.072]	No—sign reversal
Occipital	Theta	−0.094 [−0.108, −0.080]	−0.224 [−0.240, −0.209]	Yes
Occipital	Alpha	−0.180 [−0.193, −0.166]	−0.205 [−0.221, −0.190]	Yes
Occipital	Beta	−0.100 [−0.109, −0.090]	−0.129 [−0.139, −0.118]	Yes
Occipital	Gamma	−0.185 [−0.199, −0.171]	−0.101 [−0.119, −0.083]	Yes
Occipital	Total	−0.036 [−0.043, −0.028]	−0.157 [−0.166, −0.148]	Yes

## Data Availability

The original contributions presented in this study are included in the article. Further inquiries can be directed to the corresponding author.

## Abbreviations

The following abbreviations are used in this manuscript:

AI	Asymmetry index
CT	Computed tomography
EEG	Electroencephalogram
qEEG	Quantitative encephalogram
